# Identification of novel compound heterozygous variants of the PNPLA6 gene in Oliver-McFarlane syndrome with concomitant insulin resistance

**DOI:** 10.1016/j.gendis.2025.101985

**Published:** 2025-12-14

**Authors:** Ruojun Qiu, Mingming Tang, Weifen Zhu, Binghong Wang, Wenyu Li, Yongzhen Lei, Fenping Zheng

**Affiliations:** a Department of Endocrinology, The Affiliated Sir Run Run Shaw Hospital, College of Medicine, Zhejiang University, Hangzhou, Zhejiang 310016, China; b Department of Endocrinology, Jiading District Central Hospital Affiliated Shanghai University of Medicine & Health Sciences, Shanghai 201899, China; c Department of Ophthalmology, The Affiliated Sir Run Run Shaw Hospital, College of Medicine, Zhejiang University, Hangzhou, Zhejiang 310016, China; d Department of Neurology, The Affiliated Sir Run Run Shaw Hospital, College of Medicine, Zhejiang University, Hangzhou, Zhejiang 310016, China

Oliver-McFarlane syndrome (OMCS) is a rare autosomal recessive genetic disease characterized by trichomegaly, anterior pituitary hypofunction, chorioretinal degeneration and neurological manifestations. The association between this syndrome and variants in the patatin-like phospholipase domain-containing protein 6 (*PNPLA6*, OMIM 603197) gene was first reported by Hufnagel et al in 2015.^1^ The *PNPLA6* gene encodes neuropathy target esterase (NTE), which plays a critical role in phosphatidylcholine metabolism, membrane phospholipid trafficking, and axonal integrity.^2^ PNPLA6 is expressed throughout the central nervous system and eyes during human embryonic development and is widely expressed in adult tissues. Mutations in the *PNPLA6* gene alter NTE enzyme activity, thus leading to a spectrum of diseases.^3^ In this study, we reported a 17-year-old female patient who presented with absent secondary sexual characteristics during puberty with long and curly eyelashes, unexpectedly accompanied by marked insulin resistance and fatty liver disease. The patient was finally diagnosed with OMCS based on the whole exome sequencing (NGS) identifying novel compound heterozygous variants in the *PNPLA6* gene.

The patient presented with primary amenorrhea and first visited the hospital at age 14, with follow-up visits at ages 16 and 17. At age 11, she experienced significant weight gain accompanied by acanthosis nigricans on the neck, armpits and inner thighs. At age 14, she weighed 60 kg (+1SD to +2SD) and was 152 cm tall (–2SD to –1SD), with absent secondary sexual characteristics. At age 17, she weighed 66 kg with a height of 157 cm (BMI 26.8 kg/m^2^; genetically projected height range 157–167 cm [FPH formula, paternal 172 cm, maternal 159 cm]), with a round face, a cervical fat pad but no significant central obesity, Tanner stage 3 breast development, sparse pubic/axillary hair and a prepubertal vulva. No family history of metabolic disorders was reported. Notably, she had trichomegaly (curled eyelashes, Fig. S1A). The comprehensive ophthalmic examination, including pigmentation assessment and electroretinography (ERG), was normal (Fig. S1B). Neurological examination indicated bilateral horizontal nystagmus, with normal muscle strength, muscle tone, tendon reflexes and coordination. During her hospitalization from age 14 to 17, laboratory tests revealed several times that the levels of estrogen, progesterone, follicle-stimulating hormone (FSH), and luteinizing hormone (LH) and insulin-like growth factor 1 (IGF-1) were low and free thyroid hormones gradually decreased. An oral glucose tolerance test (OGTT) indicated impaired glucose tolerance and severe insulin resistance. An insulin hypoglycemia stimulating test (intravenous injection of 40 IU insulin, 0.61 IU/kg) indicated growth hormone deficiency with a normal cortisol response (Table S1). The GnRH (triptorelin) stimulation test failed to induce FSH and LH responses. The patient demonstrated delayed bone age (Fig. S1C), small uterine volume (Fig. S1D), fatty liver (Fig. S1E), and pituitary hypoplasia (Fig. S1F). We then conducted pedigree analysis. Peripheral venous blood samples were collected from the patient, her parents and sister. NGS detected novel compound heterozygous mutation of *PNPLA6* gene (NM_006702.5) in the proband. Mutation 1 (c.1697+3A > G in intron 17) was inherited from her mother; mutation 2(c.3265T > C; p. Tyr1089His in exon 30) was inherited from her father, and mutation 3 (c.2822 + 17C > T in intron 26) was also identified. Her sister does not carry either mutation of 1 or 2 (Fig. 1A and B). Pathogenicity prediction of mutation 2 was performed using protein function prediction software: Polyphen-2 (possibly damaging) and Mutation Taster (pathogenic) (Fig. S1G). Species conservation analysis of the Tyr1089 site in the human *PNPLA6* gene showed high conservation across different species during evolution (Fig. S1H). Pathogenicity and splicing alteration prediction of mutation 1 and mutation 3 was performed using software: RNA Splicer (RDDC) and SpliceAI predicted the mutation 1 was possibly likely pathogenic and caused splicing alteration, whereas mutation 3 was possibly likely benign without splicing alterations (Fig. S1I). After age 16, estrogen replacement therapy was initiated regularly and lifestyle interventions, and metformin were prescribed to improve insulin resistance. However, menstruation did not occur naturally at age 17, so artificial menstrual induction was started. Currently, she received thyroid hormone supplementation.

**Figure 1:**
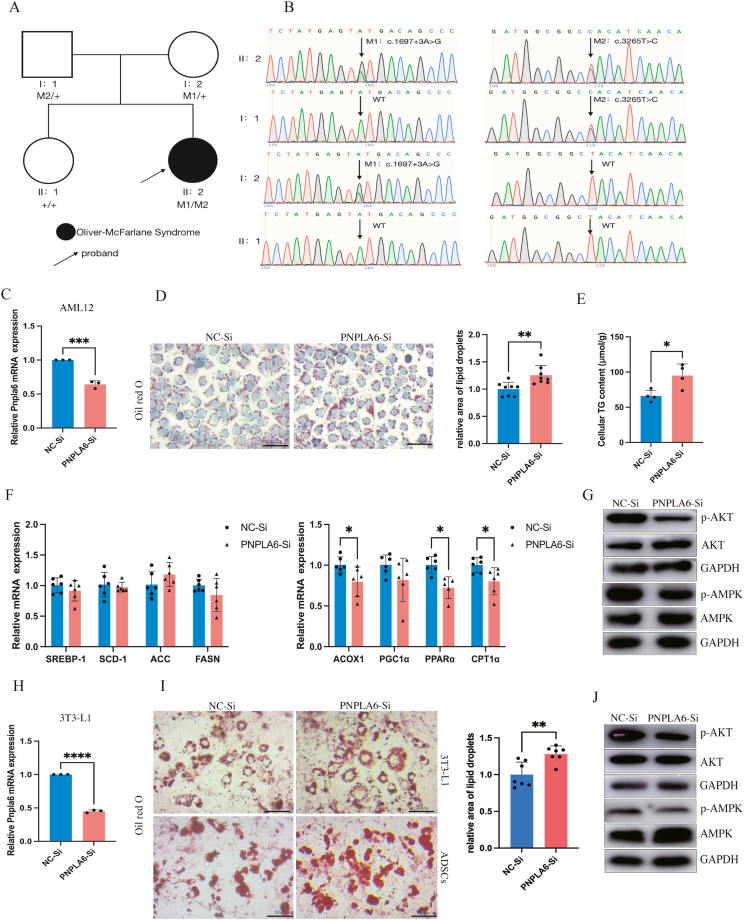
A novel compound heterozygous variants of the PNPLA6 gene in Oliver-McFarlane syndrome. **(A)** Pedigree analysis of the gene mutation. **(B)** Sanger sequencing results. M1 shows c.1697+3A > G, intron17, and M2 shows c.3265T > C (p. Tyr1089His), exon 30. + indicates wild-type allele. (**C)** Relative PNPLA6 mRNA expression in AML12 hepatocytes after siRNA transfection. **(D)** Representative images of Oil Red O staining of AML12 hepatocytes after siRNA transfection and free fatty acid (FFA) treatment (0.5 μM for 12 h, scale bars: 200 μm). The relative area of lipid droplets was analyzed via ImageJ. **(E)** Quantification of triglyceride (TG) content in AML12 cells after siRNA transfection and FFA treatment. **(F)** mRNA expression levels of key genes involved in fatty acid synthesis and oxidation in AML12 hepatocytes after siRNA transfection and FFA treatment. **(G)** Representative Western blot analysis of AKT and AMPK in AML12 cells after siRNA transfect and FFA treatment. **(H)** Relative PNPLA6 mRNA expression in 3T3-L1 cells after siRNA transfect. **(I)** Representative images of Oil Red O staining of 3T3-L1 and ADSCs after siRNA transfection and adipogenic differentiation(scale bars: 200 μm). **(J)** Representative Western blot analysis of AKT and AMPK in 3T3-L1 cells after siRNA transfection and adipogenic differentiation. Data are presented as mean ± standard deviation (SD). ∗*P* < 0.05; ∗∗*P* < 0.01; ∗∗∗*P* < 0.001.

So far, 37 cases of OMCS have been published. The clinical manifestations were variable with most patients presenting trichomegaly, short stature, retinopathy, ataxia and pituitary hormone deficiencies. Low birth weight, delayed bone age, abnormal hair, and ataxia were reported in more than half of the cases. Our patient presented with short stature, delayed bone age, trichomegaly, ocular nystagmus and pituitary hormone deficiency. Genetically, most OMCS individuals described in the literature carry compound heterozygous mutations in *PNPLA6* (mostly biallelic exonic point mutations), with only one case harboring a single variant. Our case was identified as harboring a pathogenic exon variant along with two intron mutations—a previously unreported pattern. The exon mutation site was located in the patatin-like domain, which may result in decreased NTE hydrolase activity and abnormal phospholipid metabolism, leading to the disruption of membrane integrity. One of the intron mutation sites (c.1697+3A > G, intron 17) might disrupt the donor splice site at the exon-intron junction, leading to aberrant RNA splicing. The patient's relatively mild neurological involvement and absence of retinopathy may be attributed either to the mutation pattern or the relatively short duration of the disease. However, a clear genotype–phenotype correlation requires further in-depth research in the future.

Uniquely, our patient was overweight and markedly insulin resistant, presenting with acanthosis nigricans, markedly elevated insulin levels during OGTT, and confirmed by an abnormally high dose of insulin in the insulin hypoglycemia stimulating test. In previous literature, metabolic assessments of most patients were limited, with only three patients reported to exhibit obesity or abdominal obesity. To date, no studies have linked *PNPLA6* mutations to insulin resistance or other metabolic disorders. To investigate the potential association between PNPLA6 and metabolic disorders, we silenced the *PNPLA6* gene with siRNA in different cell lines. In AML12 hepatocytes, *PNPLA6* knockdown (PNPLA6-Si) (Fig. 1C) with free fatty acid (FFA) treatment exhibited more significant lipid droplet accumulation (Fig. 1D) and higher intracellular triglyceride (TG) content (Fig. 1E) compared to control cells (NC–Si). The expression levels of fatty acid synthesis genes were unchanged, while oxidation genes, including *ACOX-1*, *CPT1α* and *PPARα* were downregulated (Fig. 1F). Concurrently, the key insulin signaling pathways, including phosphorylated Akt (p-Akt) and phosphorylated AMPK (p-AMPK) were both decreased (Fig. 1G). Similarly, siRNA-mediated *PNPLA6* knockdown in 3T3-L1 preadipocyte and adipose-derived stem cells preadipocytes (ADSCs) displayed markedly increased lipid droplet accumulation upon adipogenic differentiation (Fig. 1H and I), along with the decreased levels of p-Akt and p-AMPK (Fig. 1J).

The PNPLA protein family comprises nine mammalian members which share a conserved patatin domain conferring lipolytic/acyltransferase activity critical for lipid metabolic regulation. Specifically, PNPLA3 plays a key role in energy mobilization and fat storage, while PNPLA7 displays nutrient-responsive roles in lipid droplet maturation.^4^^,^^5^ Our preliminary data demonstrate the potential role of PNPLA6 in lipid metabolism by regulating lipid accumulation in hepatocytes or fat storage in adipocytes, accompanied by inhibition of insulin signaling. However, further *in vivo* studies targeting PNPLA6 in a tissue-specific manner are needed to fully elucidate the systemic phenotypes of PNPLA6. These findings call for clinicians (ophthalmologists, psychiatrists and endocrinologists) to pay more attention to metabolic comorbidities in PNPLA6-associated disorders and the role of PNPLA6 in metabolic regulation.

In summary, we report a novel gene mutation that induces OMCS in a patient and her pedigree. By highlighting the prominent insulin resistance in this patient, we propose and preliminarily validates, for the first time, the role of PNPLA6 in regulating lipid metabolism and insulin resistance.

## CRediT authorship contribution statement

**Ruojun Qiu:** Writing – original draft, Investigation, Data curation. **Mingming Tang:** Formal analysis, Data curation. **Weifen Zhu:** Formal analysis, Data curation. **Binghong Wang:** Formal analysis, Data curation. **Wenyu Li:** Formal analysis, Data curation. **Yongzhen Lei:** Formal analysis, Data curation. **Fenping Zheng:** Writing – review & editing, Writing – original draft, Supervision, Project administration, Methodology, Funding acquisition.

## Ethics declaration

The research protocol was approved by ethics committee of Sir Run Run Shaw Hospital affiliated with Medical School of Zhejiang University (Sir Run Run Shaw Hospital Ethics Approval 2025 Research No. 1113). This study was conducted in accordance with the ethical principles of the Declaration of Helsinki. Written informed consents were obtained from the patient and her family.

## Funding

This work was supported by the 10.13039/501100004731Natural Science Foundation of Zhejiang Province, China (No. LHDMY23H070005). The contents of the published material are solely the responsibility of the individual authors and do not reflect the view of any of the funding bodies.

## Conflict of interests

The authors declare that they have no conflict of interests.

## Figures and Tables

**Figure S1:**
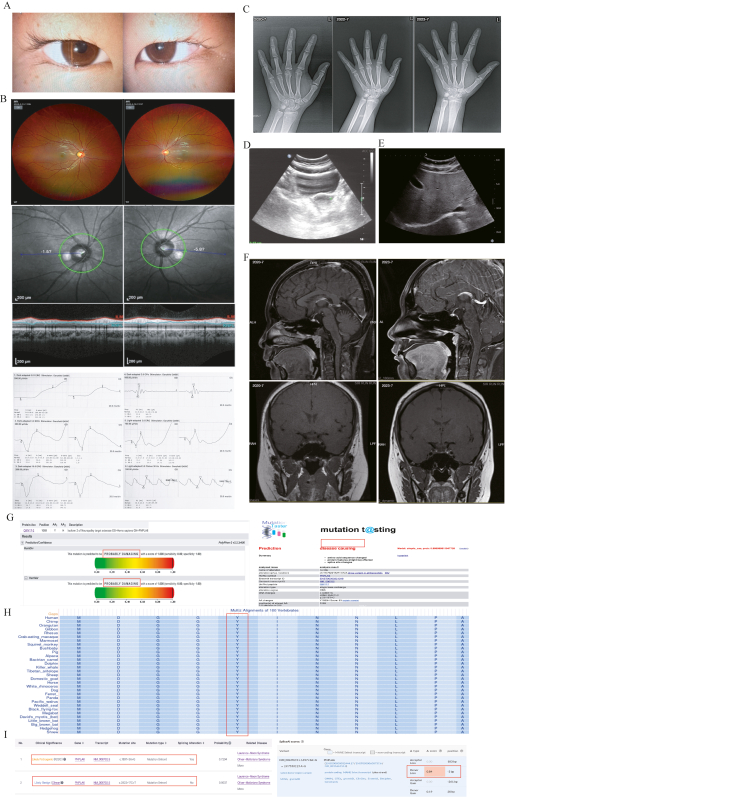
Comprehensive analysis of patients' imaging studies and biological characterization of mutated gene. **(A)** Patient's ocular appearance showing long and curled eyelashes. **(B)** Normal color fundus photography, optical coherence tomography (OCT) and electroretinography (ERG) in both eyes. **(C)** Left hand bone age X-rays of the patient at ages 14 (bone age: 11 years), 16 (bone age: 12 years), and 17 years (bone age: 14.5 years). **(D)** Uterine ultrasound of the patient at 17 years (b, 3.97 × 2.24 × 3.13 cm). **(E)** Liver ultrasound of the patient at 17 years. **(F)** Pituitary MRI of the patient at ages 14 and 17 years. **(G)** Bioinformatics analysis of the PNPLA6 exon mutation. Results of cores of PNPLA6 c.3265T > C (p.Tyr1089His) exon30 in Polyphen-2 and Scores of damaging mutations in Mutation Taster. **(H)** Results from amino acid sequence alignments of Tyr1089 in PNPLA6 orthologues generated through the UCSC Genome Browser. **(I)** Pathogenicity and RNA splicing analysis of the PNPLA6 intron mutation using RDDC and SpliceAI.

## Appendix A Supplementary data

Multimedia component 1 Multimedia component 1

Table S1 Detailed results of related experimental tests at ages 14 and 17 years. Table S1

## References

[1] Hufnagel R.B., Arno G., Hein N.D. (2015). Neuropathy target esterase impairments cause Oliver-McFarlane and laurence-moon syndromes. J Med Genet.

[2] Kretzschmar D. (2022). PNPLA6/NTE, an evolutionary conserved phospholipase linked to a group of complex human diseases. Metabolites.

[3] Liu J., Hufnagel R.B. (2023). PNPLA6 disorders: what's in a name?. Ophthalmic Genet.

[4] Moon S., Eun C., Kyung J. (2022). A PNPLA3 polymorphism confers lower susceptibility to incident diabetes mellitus in subjects with nonalcoholic fatty liver disease. Clin Gastroenterol Hepatol.

[5] Yang A., Mottillo E.P., Mladenovic-Lucas L., Zhou L., Granneman J.G. (2019). Dynamic interactions of ABHD5 with PNPLA3 regulate triacylglycerol metabolism in brown adipocytes. Nat Metab.

