# Supplementary material for: Identification of novel compound heterozygous variants of the PNPLA6 gene in Oliver-McFarlane syndrome with concomitant insulin resistance
Source: Genes Dis. 2025 Dec 14;13(5):101985. doi: 10.1016/j.gendis.2025.101985 (PMC13123492; doi:10.1016/j.gendis.2025.101985)
Supplement: Multimedia component 1 [file mmc1.docx]

**Supplementary Materials & Methods**

**Ethical Approval**

We included a young female patient diagnosed with OMCS who received multiple hospitalizations for diagnosis and treatment from 2020 to 2023 at the Department of Endocrinology, Sir Run Run Shaw Hospital, Zhejiang University School of Medicine, and her family members of parents and sister. This study was conducted in accordance with the ethical principles of the Declaration of Helsinki. This study was approved by the institutional ethics committee of Sir Run Run Shaw Hospital, and informed consent was obtained from the patient and her family members.

**Clinical Evaluations**

Detailed medical history inquiry and physical examination were conducted on the patient. Endocrine tests were completed in serum samples isolated from whole blood, including sex hormones, thyroid hormones, growth hormone (GH) and adrenocorticotropic hormone (ACTH). Oral glucose tolerance tests (OGTT), insulin hypoglycemia stimulating test and GnRH (Triptorelin) stimulation test were performed. Routine funduscopic examination was used to examine the patient’s retina. Magnetic resonance imaging (MRI), uterine ultrasound and bone age assessment were also completed.

**Whole-exome sequencing**

After obtaining informed consent, 5 ml of peripheral blood was collected from the proband, her parents, and her sister; Genetic testing was performed by Di'an Diagnostic Group Technology Co., Ltd.

**Bioinformatics Analysis**

The UCSC database were used to show the multiple amino acid alignments of PNPLA6 orthologues (https://genome-asia.ucsc.edu). Protein function prediction software including PolyPhen2 (http://genetics.bwh.harvard.edu/pph2/) and Mutation Taster (http:// www.mutationtaster.org/) were used to predict the pathogenicity of the identified variants. Pathogenicity and splicing alteration prediction of intron mutations was performed using software: RNA Splicer (RDDC, https://rddc.tsinghua-gd.org/tool) and SpliceAI (https://spliceailookup.broadinstitute.org).

**Cell culture and treatments**

AML12 and 3T3- L1 cells were obtained from American Type Culture Collection and incubated in a humidified incubator at 37 °C with 5% CO2 at 37 °C under 5% (v/v) CO2 and 95% humidity. AML12 cells were grown in DMEM/F12 supplemented with 10% (v/v) FBS (SERANA; S-FBS-EU-015), 0.1 μM dexamethasone (D4902; Sigma-Aldrich), insulin-transferrin-selenium (I3146; Sigma-Aldrich) and 1% penicillin-streptomycin. 3T3-L1 cells were cultured in DMEM with 10% (v/v) newborn calf serum (Gibco, 16010159) and 1% IU/ml penicillin/streptomycin. ADSCs were isolated from mice inguinal adipose tissue, digested with collagenase type I (0.75% w/v, 37 °C, 60min) to dissociate the extracellular matrix, then filtered through 70μm mesh, and plated in culture flasks with growth medium (DMEM with 10% (v/v) FBS and 1% IU/ml penicillin/streptomycin). After 24hours, non-adherent cells are discarded, and adherent ADSCs are expanded through serial passaging for downstream applications. SiRNA sequence sense 5’-GCCUGUGAAUAUAGCUACUTT-3’ and antisense 5’-AGUAGCUAUAUUCACAGGCTT-3’. Seed cells in 24-well plates (1–2×10⁵ cells/well) in complete medium and incubate overnight. Mix 2 μL 20 μM siRNA and 2 μL transfection reagent in 100 μL serum-free medium and incubate 15 min at RT then add complexes dropwise to cells covered with 400 μL serum-free medium. After 6 h incubation replace with complete medium and analyze knockdown at 48-72h. To induce steatosis, 0.75 mM FFA (palmitic acid: oleic acid, 1:2) was applied to AML12 cells for 12 hours. 3T3­ L1 cells were induced to differentiate into adipocytes by classic adipogenic cocktail comprising insulin, dexamethasone, and IBMX.

**Quantitative real-­ time reverse transcriptase (qRT)­PCR**

Total RNAs were isolated from liver and iWAT samples using Trizol regent (Accurate Biotechnology, AG21101) and then reverse transcribed to cDNA using a Hifair II First Strand cDNA Synthesis Kit (Yeasen, China) and PCR was performed using Hieff quantitative PCR SYBR Green Master Mix (Yeasen, China). The relative expression level of each gene was normalized to the level of GAPDH and calculated by 2−ΔΔCT method. The primer sets were listed as following. *GAPDH* F: 5’- ATGAC ATCAA GAAGG TGGTG AAGC- 3’, R: 5’- GAAGA GTGGG AGTTG CTGTT GAAG-3’; *PNPLA6* F: 5’-TCTATGGCCGGAAGATTATGCG, R:5’-TGTAG GGTCG GAGTC TCCTT T-3’. *ACC* F: 5’- GCAGC AGTTA CACCA CAT-3’, R: 5’-CGCCA TCTTC CACAA TATAC-3’; *SREBP-1c* F: 5’­ CAAGG CCATC GACTA CATCC G-3’, R: 5’-CACCA CTTCG GGTTT CATGC-3’; *ACOX-1* F: 5’­CCGCC ACCTT CAATC CAGAG-3’, R: 5’-CAAGT TCTCG ATTTC TCGAC GG-3’; *CPT1a* F: 5’-CTCCG CCTGA GCCAT GAAG-3’, R: 5’-CACCA GTGAT GATGC CATTC T-3’; *PGC-1α* F: 5’-GGACA TGTGC AGCCA AGACT CT-3’, R: 5’-CACTT CAATC CACCC AGAAA GCT-3’; *PPARα* F: 5’- GCAGT GCCCT GAACA TCGA-3’, R: 5’­ CGCCG AAAGA AGCCC TTAC-3’.

**Western blotting**

RIPA buffer mixed with 100 mM phenylmethanesulfonyl fluoride was used to lyse cell pellets or tissues (FD0100; Fude Biological Technology). With the help of Bicinchoninic acid analysis, total protein concentration was determined (FD2001; Fude Biological Technology). On an SDS-polyacrylamide gel, protein samples were separated, transferred to PVDF membranes, and then incu- bated with primary antibodies. Antibodies specific for mTOR (2983), p-mTOR (5536), AMPK (5832), p-AMPK (2535), AKT (9272), P-AKT (4060) were obtained from Cell Signaling Technology. HRP goat anti-rabbit IgG (AS014; ABclonal). HRP goat anti-mouse IgG (AS003; ABclonal). The protein bands were analyzed using Image J with GAPDH as the internal control.

**Oil red O- staining**

Oil Red working solutions (Jiancheng Nanjing, D027) were filtered through 0.2-mm-pore-diameter filters. Cells were incubated with Oil Red O working solution for 15 min and washed several times with PBS according to the manufacturer's instructions and inspected under a light microscope (Olympus).

**Cellular TG Quantification**

Intracellular triglyceride (TG) levels were quantified per Cellular Triglyceride Enzymatic Assay Kit protocol (Applygeng, E1013). Briefly, harvested cells were lysed, and the lysates were incubated at 70°C for 10 minutes. After centrifugation, the supernatant was subjected to enzymatic analysis for TG measurement.

**Statistical analyses**

GraphPad Prism 9 was used to analyze the values in this study. All results are displayed as the mean ± SD. The comparisons between the 2 groups were made using unpaired Student t tests. A p-value < 0.05 was considered statistically significant (*p < 0.05, **p < 0.01, ***p < 0.001).
