# Supplementary material for: Identification of novel compound heterozygous variants of the PNPLA6 gene in Oliver-McFarlane syndrome with concomitant insulin resistance
Source: Genes Dis. 2025 Dec 14;13(5):101985. doi: 10.1016/j.gendis.2025.101985 (PMC13123492; doi:10.1016/j.gendis.2025.101985)
Supplement: Table S1 — Detailed results of related experimental tests at ages 14 and 17 years. [file mmc2.docx]

Table S1. The detailed results of related experimental tests (14y and 17y)

| GnRH(triptorelin)  stimulation test (14y) | baseline | | | | | 1 h | | | | |
| --- | --- | --- | --- | --- | --- | --- | --- | --- | --- | --- |
| LH (IU/L) | 0.08 | | | | | 0.11 | | | | |
| FSH (IU/L) | 0.04 | | | | | 0.10 | | | | |
| Clonidine test (14y) | Baseline | 0.5h | | 1h | | | 1.5h | | | 2h |
| GH (μg/L) | 0.1 | 0.17 | | 0.36 | | | 0.26 | | | 0.33 |
| Insulin hypoglycemia stimulating test (17y) | 0min | 15 min | 30 min | | | 45 min | 60 min | | 75 min | 90 min |
| Glucose (mmol/L) | 5.43 | 3.68 | 2.79 | | | 2.2 | 1.8 | | 2.44 | 2.98 |
| Cortisol (μg/dL) | 7.48 | 5.53 | 5.43 | | | 5.86 | 9.27 | | 17.18 | 20.97 |
| ACTH (ng/L) | 10 | 6 | 9 | | | 12 | 19 | | 48 | 49 |
| GH (μg/L) | 0.2 | 0.1 | 0.1 | | | 0.2 | 0.2 | | 0.2 | 0.2 |
| OGTT (17y) | 0h | 1h | | | 2h | | | 3h | | |
| Glucose  (mmol/L) | 4.77 | 9.1 | | | 10.79 | | | 7.24 | | |
| C-peptide  (pmol/L) | 1656 | 5041 | | | 7584 | | | 6868 | | |
| Insulin  (uIU/ml) | 29.79 | 220.26 | | | >300 | | | >300 | | |
| GnRH (triptorelin) stimulation test  (17y) | baseline | | | | | 1 h | | | | |
| LH (IU/L) | 0.02 | | | | | 0.01 | | | | |
| FSH (IU/L) | 0.1 | | | | | 0.08 | | | | |

GnRH, Gonadotropin-Releasing Hormone ; LH, luteinizing hormone; FSH, follicle-stimulating hormone; GH, Growth Hormone; ACTH, adrenocorticotropic hormone; OGTT, oral glucose tolerance test.
